# HEART: heart exercise and remote technologies: A randomized controlled trial study protocol

**DOI:** 10.1186/1471-2261-11-26

**Published:** 2011-05-31

**Authors:** Ralph Maddison, Robyn Whittaker, Ralph Stewart, Andrew Kerr, Yannan Jiang, Geoffrey Kira, Karen H Carter, Leila Pfaeffli

**Affiliations:** 1 Clinical Trials Research Unit, University of Auckland, Morrin Road, Glen Innes, Auckland 1121, New Zealand; 2 Department of Medicine, University of Auckland, Grafton Road, Auckland, 1010, New Zealand; 3 Epidemiology and Biostatistics, University of Auckland, Morrin Road, Glen Innes, Auckland 1072, New Zealand

## Abstract

**Background:**

Cardiovascular disease (CVD) is the leading cause of death worldwide. Cardiac rehabilitation (CR) is aimed at improving health behaviors to slow or reverse the progression of CVD disease. Exercise is a central element of CR. Technologies such as mobile phones and the Internet (mHealth) offer potential to overcome many of the psychological, physical, and geographical barriers that have been associated with lack of participation in exercise-based CR. We aim to trial the effectiveness of a mobile phone delivered exercise-based CR program to increase exercise capacity and functional outcomes compared with usual CR care in adults with CVD. This paper outlines the rationale and methods of the trial.

**Methods:**

A single-blinded parallel two-arm randomized controlled trial is being conducted. A total of 170 people will be randomized at 1:1 ratio either to receive a mHealth CR program or usual care. Participants are identified by CR nurses from two metropolitan hospitals in Auckland, New Zealand through outpatient clinics and existing databases. Consenting participants are contacted to attend a baseline assessment. The intervention consists of a theory-based, personalized, automated package of text and video message components via participants' mobile phones and the Internet to increase exercise behavior, delivered over six months. The control group will continue with usual CR. Data collection occurs at baseline and 24 weeks (post-intervention). The primary outcome is change in maximal oxygen uptake from baseline to 24 weeks. Secondary outcomes include post-intervention measures on self-reported physical activity (IPAQ), cardiovascular risk factors (systolic blood pressure, weight, and waist to hip ratio), health related quality of life (SF-36), and cost-effectiveness.

**Discussion:**

This manuscript presents the protocol for a randomized controlled trial of a mHealth exercise-based CR program. Results of this trial will provide much needed information about physical and psychological well-being, and cost-effectiveness of an automated telecommunication intervention. If effective, this intervention has enormous potential to improve the delivery of CR and could easily be scaled up to be delivered nationally (and internationally) in a very short time, enhancing the translational aspect of this research. It also has potential to extend to comprehensive CR (nutrition advice, smoking cessation, medication adherence).

**Trial Registration:**

ACTRN12611000117910

## Background

An estimated 16.7 million or 29% of total global deaths result from the various forms of cardiovascular disease (CVD). The majority of deaths were either due to ischemic heart disease or stroke [1]. By 2020 the global burden of fatal and non-fatal CVD is anticipated to grow in importance in both absolute and relative terms [1]. Approximately 68 million deaths occur worldwide of all causes, of which approximately 25 million (37%) deaths will be due to cardiovascular causes, with ischemic heart disease accounting for about 11 million deaths [1]. The total number of disability affected life years (DALYs) attributable to CVD is expected to rise from 134 million in 1990 to about 204 million in 2020. Ischemic heart disease is expected to be the leading cause of DALYs in 2020 (82 million DALYs) [1].

Cardiac rehabilitation (CR) is an essential component of secondary prevention of CVD, and is a complex intervention that may include a variety of therapies, including risk factor education, psychological therapy, and medication [2]. International guidelines consistently identify exercise therapy as a central element of CR [3-6]. Exercise-based CR have been shown to be cost-effective for those who participate [7].

A Cochrane systematic review of exercise-based CR reported that all-cause mortality was reduced by 27% (OR 0.73, 95% CI 0.54 to 0.98) with exercise-only rehabilitation compared with usual care [2]. The reduction in CVD mortality was 31% (random effects model OR 0.69, 95% CI 0.51 to 0.94) with exercise-only rehabilitation [2]. These findings have been supported by a separate meta-analysis [8].

There are numerous benefits associated with exercise training in patients with CVD, including improvement in exercise tolerance as assessed by both exercise duration and oxygen uptake (VO_2_), peak cardiac output, peak cardiac power output, and overall cardiac reserve [9-12]. Exercise is also associated with a constellation of changes such as improvements in lipid profile [2], insulin sensitivity, reduction in BMI, percentage body fat, reduction in inflammatory variables, and improvement in autonomic tone [12].

Despite the documented benefits of CR, provision is inadequate in all countries in which it has been measured [13]. In New Zealand, uptake of CR is only 20% with high levels of dropout after enrolment [14]. International data show that adherence with exercise recommendations is low [15-17]; between 10% and 36% of individuals dropout of supervised exercise programs. Long-term exercise adherence is worse, with up to 50% of people not participating in regular exercise at 6 [18] and 18-months [16]. Those who do not adhere to exercise-based CR do not realize the benefits of regular exercise such as increased cardiovascular fitness, decreased weight, and improved lipid profile [15].

The main reasons that people give for not accepting the invitation to attend center based CR classes, which include supervised exercise--held for groups in hospitals, gymnasiums, or community centers--are problems with access and parking [3,19,20], a dislike of these groups [21], and work or domestic commitments [22-24]. Psychological barriers that have been associated with lack of participation in CR programs include the perceived benefits of rehabilitation [25,26], lack of self-motivation and self-esteem [3,17,27], and lack of social support [28]. New Zealand research has reported similar findings with lack of access to transport associated with poor attendance at CR [14].

Many of these problems can be overcome by home-based rehabilitation, which has been introduced in an attempt to widen access and participation [29]. A recent Cochrane review and meta-analysis [30] of 12 studies (n = 1938) found no differences between home-based and center-based CR in terms of mortality (relative risk 1.31, 95%CI 0.65 to 2.66), cardiac events, exercise capacity (standardized mean difference -0.11, -0.35 to 0.13), modifiable risk factors (weighted mean difference systolic blood pressure (0.58 mm Hg, -3.29 mm Hg to 4.44 mm Hg), total cholesterol (-0.13 mmol/l, -0.31 mmol/l to 0.05 mmol/l), low density lipoprotein cholesterol (-0.15 mmol/l, -0.31 mmol/l to 0.01 mmol/l), or health related quality of life. In the home-based participants, there was evidence of superior adherence; however healthcare costs of the two forms of CR were similar. While these findings are encouraging and support the use of home-based cardiac rehabilitation, data from the United Kingdom suggest that only about 20% of CR programs currently offer an evidence-based home option [30].

In terms of increasing physical activity, telephone-based interventions can be effective [31,32]; however mobile phones promise an even more effective way to reach many people who seldom access CR services. Mobile phones are carried by most adults most of the time. Mobile health or mHealth programs, which include mobile phones and the Internet, can be delivered anywhere at any time, removing geographical barriers to CR. Communication with participants can be regular and frequent, and messages can be delivered in a time-sensitive or time-appropriate way to support behavior change in a more continuous manner. Programs delivered by mobile phone do not require participants to attend a clinic and could be used as an alternative option for people that find it difficult to attend exercise sessions. This makes programs far more proactive (initiated by the service) rather than requiring action by the participant before they can start to impart information or provide support. Although automated, the electronic nature of the program means it can be tailored to specific cultural or age groups, and health needs.

This paper presents the rationale and methods of a randomized controlled trial to investigate the effects of an mHealth delivered exercise-based CR program to improve exercise capacity and functional outcomes in people with CVD.

## Method/Design

The study design is a single-blinded two-arm parallel randomized controlled trial comparing an mHealth exercise-based program of CR to usual CR alone.

### Aims

The primary aim is to investigate the effects of an mHealth delivered exercise-based CR program to increase exercise capacity compared with usual care (exercise advice and an offer to participate in a cardiac club) in New Zealand adults with a diagnosis of CVD. Secondary aims are to examine the effect of the intervention on physical activity, systolic blood pressure, weight, waist to hip ratio, health related quality of life, and cost effectiveness.

### Study sample

Eligible participants are adults aged 18 years or more with a clinically documented diagnosis of ischemic heart disease (angina, myocardial infarction, revascularization, including angioplasty, stent or coronary artery bypass graft) within the previous three to twelve months. Participants must be current outpatients who are clinically stable, able to perform exercise, can understand and write English, own a mobile phone, and have access to the Internet. Participants who have been admitted to hospital with heart disease within the previous six weeks, have terminal cancer, or have significant exercise limitations other than CVD are excluded.

Eligible participants are identified by research nurses from two metropolitan hospitals through outpatient clinics and existing databases. Research nurses contact potential participants to determine their interest in the trial. Those agreeing to participate are screened for eligibility and sent a study pack, which includes a participant information sheet and consent form. Contact details of interested participants are sent to the research team. After one week, participants are telephoned to confirm their interest in the study and schedule a baseline assessment.

### Sample size calculation

The target sample size of 170 participants (85 per group) will provide 90% power at 5% level of significance (two-sided) to detect a treatment difference of at least 2.5 ml^-1^.kg.min^-1 ^between the two groups, on change in VO_2max _from baseline to 24 weeks assuming a standard deviation of 5 ml^-1^.kg.min^-1^. Recruiting at least 50 Māori (indigenous) participants (25 per arm) will provide 80% power to detect a treatment difference of 4.0 ml.kg^-1^.min^-1 ^between the two groups under the same assumptions.

### Ethics approval

Ethical approval for the trial was received from the Northern X Regional Ethics Committee (NTX/10/10/099). Approval was also obtained from the two Metropolitan Hospitals' respective Ethics Approval Committees.

### Randomization and blinding

Participants are randomly allocated to either the control (usual care) or intervention (mHealth) arms at a 1:1 ratio using a computerized stratified (sex, ethnicity, and adherence to Phase 2 CR) minimization [33] sequence. Ethnicity is based on self-identification as Māori or non-Māori. Adherence to CR is classified as attending at least one community CR session.

The allocation sequence is overseen by the project statistician (YJ). Assessors of the primary outcomes will be blinded to treatment allocation; however it is not possible to blind participants to their allocation.

### Intervention

The intervention is a personalized automated program of SMS (short messaging service) text messages delivered over six months via mobile phone and supported with a personalized website that participants can log on to. This includes personalized feedback on progress against goals, as well as information on various forms of exercise, and links to other websites (e.g. local exercise programs and cardiac clubs), as well as CR related information.

The goal of the program is to have individuals participate in moderate to vigorous aerobic-based exercise for a minimum of 30 minutes (preferably more) on most days (at least 5) of the week, in line with current recommendations [34], and American College of Sports Medicine (ACSM) guidelines [35]. Consistent with recommendations for supervised home-based exercise [36] the program involves regular exercise prescription, technical support, and provision of behavioral strategies (goal setting, exercise scheduling and overcoming barriers) to increase exercise adherence, delivered via mobile phone. The intervention also focuses on altering the key mediators of behavior change, including self-efficacy, social support, intention and motivation. Specific details are provided below.

### Exercise prescription

A core component of the program involves a prescribed regimen of individualized exercise based on personal preferences and participant's current levels of fitness. A baseline exercise test forms the basis for subsequent exercise prescription. ACSM guidelines for exercise in cardiac patients are followed [35]. The prescribed exercise intensity is sufficient to induce a "training effect", yet below a metabolic load that evokes abnormal clinical signs or symptoms. Exercise duration is increased according to symptoms and clinical status. Exercise intensity is increased gradually as tolerated [15]. Criteria for progression is according to the following order: duration, frequency, then intensity [10]. Participants are taught and encouraged to use ratings of perceived exertion (RPE) to achieve the desired intensity. An equivalent RPE level of 11 to 13 (6-20 scale) ("fairly light" and "somewhat hard") [37] is used during the early stages and 13 to 15 ("somewhat hard" to "hard") in the latter stages [10].

A recent systematic review concluded that interventions at the level of the individual incorporating components of brief advice, supported use of pedometers, and telecommunications can encourage people to walk more [15]. Hence these components are included in this intervention. Walking is the primary (default) mode of activity but other activities such as swimming and cycling are encouraged based on individual preferences, past history, and access to existing resources (e.g., swimming pool, gym). A pedometer is provided to participants in the intervention group and step counts are used to indicate volume of activity for each given week. SMS messages are sent to participants outlining their prescribed exercise for each week, including duration, frequency, and intensity of exercise. Contact is more intensive during the exercise initiation period (12-weeks).

### Support and behavior change strategies

Participants also receive SMS messages focused on altering key mediators of physical activity to facilitate adherence to exercise behavior. Messages include strategies on overcoming barriers to be active, increasing motivation to exercise, scheduling exercise into one's daily routine, and goal setting. The behavioral support program is grounded in self-efficacy theory [20,38-40], which is a key psychosocial determinant of adherence to exercise and physical activity [18,41,42].

### Internet support

A website is also available for participants to access, which allows participants to retrieve on demand any of the messages sent to their phones. Video messages, motivational messages, and weekly health and exercise tips are also included. Using the website, participants can document and monitor their physical activity progress, set goals, identify barriers to exercise and generate solutions to overcome these. Participants can also view role model videos and access other CR information. For example, regular postings are added to the website encouraging participants to access existing community-based programs, for those interested.

An important component of the intervention is the use of role modeling, which refers to the process of learning behaviors from viewing other people's behaviors and the outcomes of that behavior [39,40]. Role modeling is an important source of self-efficacy and is one of the primary modes used by individuals to gain socialization information and cognitive skills [39,40].

During the intervention, participants can access brief (30 - 120 sec) videoed vignettes of other cardiac patients discussing their experiences of exercise, the types of problems they faced, how they coped, and any advice they can offer. Consistent with theory and to enhance the motivational properties of the videos, a variety of different models are used to cover general age, gender, and ethnic groups. Other vignettes from cardiologists, cardiac rehabilitation nurse specialists, and exercise physiologists are included, which provide information outlining the benefits of exercise, physiological responses and safety issues.

### Control group

The control group is directed to receive usual community-based CR, which currently involves encouragement to be physically active and an offer to join a local cardiac club.

### Study outcome measures

Assessments are undertaken in a physiology laboratory at the University of Auckland at baseline and 24 weeks post-randomization. The research team arranges an appointment at an agreed time to undergo assessment. To facilitate attendance at the assessments participants are offered taxi transport to and from testing, or are provided with a petrol voucher to off-set any travel costs. On arrival, the researcher explains procedures and collects the signed consent forms. Physical measurements (blood pressure, height, weight, waist and hip circumference are conducted prior to a maximal oxygen uptake treadmill test (VO_2max_) to assess exercise capacity. Following a rest, participants complete a questionnaire booklet and a six minute walk test.

### Primary outcome

The primary outcome is exercise capacity measured via maximal oxygen uptake (VO_2max_) at 24-weeks. A standardized exercise testing treadmill protocol is used to assess VO_2max _using gas analysis. A ramp protocol is initiated at a comfortable walking speed, with a gradient increase of 2 degree steps at each minute. Exercise testing follows ACSM guidelines [9] and is conducted by a trained physiologist. A medical physician is available to deal with any emergencies that might arise.

### Secondary outcomes

Weight, waist and hip circumference, and blood pressure are measured using standard procedures. The Pulsecor R6.5 suprasystolic measurement system/BPPlus is used to assess systolic and diastolic pressure as well as arterial compliance. Participant's height is measured to the nearest 0.1 cm using a stadiometer and weight to the nearest 0.1 kilograms using electronic scales. Body mass index is derived from the weight divided by height (m) squared. Waist circumference is measured using a flexible tape measure placed around the participant's waist at the level of the umbilicus. Hip circumference is measured using an anthropometric measuring tape placed around the furthest protrusion of the buttocks as seen from a lateral perspective. Waist-hip ratio calculated according to ISAK protocols [43].

Psychological variables include self-efficacy (situational self-confidence) to exercise and motivations are assessed. Task self-efficacy is assessed using a scale adapted from the Self-Efficacy Scale [44]. Participants rate their confidence to perform physical activities for increasing periods of time (i.e., 10, 30, and 60 min) at three intensities (i.e., easy, moderate, and hard). A key is provided to define the various intensity levels. Mean scores are calculated with higher values indicating greater efficacy to perform physical activity for longer duration and greater intensity.

Participants' confidence to exercise in the face of obstacles (barrier efficacy) is assessed using the Barriers Efficacy Scale [44]. Participants rate their confidence to overcome seven common reasons preventing people from participating in exercise sessions on a scale ranging from 0% (no confidence at all) to 100% (completely confident). Mean scores are calculated with higher values indicating greater efficacy to overcome barriers to exercise.

Participants' confidence to schedule exercise into their daily lives is assessed with three items [45]. Participants rate their confidence on a scale ranging from 0% (no confidence at all) to 100% (completely confident). Mean scores are calculated with higher values indicating greater efficacy to schedule exercise regularly into their daily lives.

Self-determination to exercise is operationalized using the Locus of Causality for Exercise Scale; a reliable and valid three-item self-report measure of the extent to which participants feel they choose to exercise with no sense of coercion [46].

Participants rate how much they agree or disagree with each statement on a seven-item Likert scale from 1 (Strongly disagree) to 7 (Strongly agree) indicating their motivation to perform exercise. Mean scores are calculated and higher scores indicate greater self-determination or a more internal perceived locus of causality.

Physical activity is assessed using the International Physical Activity Questionnaire, a reliable and validated 7-day recall measure which provides a comprehensive evaluation of daily physical activities, and assesses the time spent walking, and engaging in light, moderate, and vigorous-intensity activities across various domains [47].

Health related quality of life is assessed using the standard version of the Short Form SF36 version 2 [22]. The SF36v.2 measures perceived health across eight domains using a 0-100 scale: 1) Physical Functioning; (2) Role-Physical; (3) Bodily Pain; (4) General Health; (5) Vitality; (6) Social Functioning; (7) Role-Emotional; and (8) Mental Health. The items and scales are scored so that a higher score indicates a better health state. Two summary measures, physical (PCS) and mental (MCS) components, can also be calculated using the transformed 0-100 scales.

Participants complete the 6-min walk test--a reliable and valid measure of functional outcome [20], which is used by many cardiac rehabilitation programs both for initial assessment and to document functional outcomes after completion of the cardiac rehabilitation program [7]. It is simple to conduct, has excellent patient acceptance because exercise levels during the test closely resemble usual patient activities, and costs of administration are low.

Cost information, including cost of program, and direct medical costs (including cost of treatment, primary care, secondary care and over-the-counter medications) are collected for the cost-effective analysis. The EQ5D [48] is used to obtain a single preference index for calculation of Quality Adjusted Life Year (QALY) to assess cost per QALY for comparison with other CR programs. EQ-5D is a descriptive system of health-related quality of life states consisting of five dimensions (mobility, self-care, usual activities, pain/discomfort, anxiety/depression) each of which can take one of three responses. The responses record three levels of severity (no problems/some or moderate problems/extreme problems) within a particular EQ-5D dimension.

Adverse events (AE) including any illness, sign, symptom, or clinically significant abnormality that has appeared or worsened during the course of the clinical trial, regardless of causal relationship to the treatment(s) under study, are also recorded.

### Statistical analyses

Statistical analyses will be performed using SAS version 9.2 (SAS Institute Inc. Cary NC) and R version 2.12 (R Foundations for Statistical Computing), with a 5% significance level (two-sided) maintained throughout the analyses. Treatment evaluations will be performed on the principle of intention to treat (ITT), using the observed data collected from all randomized participants. Missing data in the primary outcome measure will be imputed using a prespecified approach. The analysis of covariance (ANCOVA) regression model will be used to evaluate the main treatment effect on change in VO_2max _from baseline to 24 weeks, adjusting for baseline values, age, sex, self-identified ethnicity, and CR adherence (stratification factors). A similar approach will be used for other continuous outcome measures. Logistic regression model will be considered for estimating the proportion of participants meeting physical activity recommendations. A prespecified analysis plan outlines the detailed statistical methods.

## Discussion

The mHealth intervention trial described in this paper is the first of its kind to investigate the effect on an objective outcome such as VO_2max_. Other mobile phone interventions exist for cardiac patients; however our proposed research differs considerably in the application of technology. For example, the Australian e-health research center and the CSIRO have developed a technology-based care assessment platform for CR, which is currently being trialed [49]. The care assessment platform includes video or teleconferencing with 'mentors', whereas our program is completely automated and utilizes SMS and the Internet to deliver specific theory based behavior change strategies.

Results from this research have enormous potential to improve the delivery of CR and could easily be scaled up to be delivered nationally (and internationally) in a very short time, enhancing the translational aspect of this research. It has potential to extend to comprehensive CR (nutrition advice, medication adherence). The mHealth program could be offered to cardiac patients as an adjunct to existing services and could also be exported as a delivery model for future health care programs. This research has potential to improve access to health care programs and could be used to inform future policy and health care interventions.

## List of Abbreviations

ACSM: American College of Sports Medicine; CI: 95% Confidence Interval; CR: Cardiac Rehabilitation; CVD: Cardiovascular Disease; DALYs: Disability Affected Life Years; METs: Metabolic Equivalent; mHealth: Mobile Health; OR: Odds Ratio; RPE: QALY: Quality Adjusted Life Year; RPE: Ratings of Perceived Exertion; SF-36: Short Form 36; SMS: Short Messaging Service; VO_2_: Volume of oxygen uptake; VO_2max_: Volume of Maximum Oxygen Uptake; VO_2_R: Volume of Oxygen Uptake Reserve.

## Competing interests

The authors declare that they have no competing interests.

## Authors' contributions

Principal responsibility for study design and conduct is assumed by RM. RM, RS, AK, YJ, LP, and GK contributed to the development of the intervention and study design. YJ undertook sample size calculations and designed the statistical analysis plan. LP, GK, KC are involved in the data collection and overall project management. RM drafted the manuscript. All authors read and commented on drafts and approved the final manuscript.

## Pre-publication history

The pre-publication history for this paper can be accessed here:

http://www.biomedcentral.com/1471-2261/11/26/prepub
